# Dopamine modification of glycolytic enzymes impairs glycolysis: possible implications for Parkinson’s disease

**DOI:** 10.1186/s12964-024-01478-0

**Published:** 2024-01-29

**Authors:** Bing Chen, Qian Zhang, Xiaoru Zhong, Xinwei Zhang, Xin Liu, Hongyang Wang, Fan Yang, Jingjing Zhang, Jingnan Huang, Yin-Kwan Wong, Piao Luo, Jigang Wang, Jichao Sun

**Affiliations:** 1 Shenzhen Clinical Research Center for Geriatrics and Shenzhen Institute of Respiratory Disease, Shenzhen People’s Hospital (The Second Clinical Medical College, Jinan University; The First Affiliated Hospital, Southern University of Science and Technology), Shenzhen, China; 2 Integrated Chinese and Western Medicine Postdoctoral Research Station, Jinan University, Guangzhou, China; 3 State Key Laboratory for Quality Ensurance and Sustainable Use of Dao-di Herbs, Artemisinin Research Center, and Institute of Chinese Materia Medica, China Academy of Chinese Medical Sciences, Beijing, China; 4 School of Traditional Chinese Medicine and School of Pharmaceutical Sciences, Southern Medical University, Guangzhou, China; 5 Department of Oncology, The Affiliated Hospital of Southwest Medical University, Luzhou, China

**Keywords:** Dopamine (DA), Parkinson’s disease (PD), Glycolysis, Mitochondrial dysfunction

## Abstract

**Background:**

Parkinson’s disease (PD), a chronic and severe neurodegenerative disease, is pathologically characterized by the selective loss of nigrostriatal dopaminergic neurons. Dopamine (DA), the neurotransmitter produced by dopaminergic neurons, and its metabolites can covalently modify proteins, and dysregulation of this process has been implicated in neuronal loss in PD. However, much remains unknown about the protein targets.

**Methods:**

In the present work, we designed and synthesized a dopamine probe (DA-P) to screen and identify the potential protein targets of DA using activity-based protein profiling (ABPP) technology in combination with liquid chromatography-tandem mass spectrometry (LC–MS/MS). In situ pull-down assays, cellular thermal shift assays (CETSAs) and immunofluorescence were performed to confirm the DA modifications on these hits. To investigate the effects of DA modifications, we measured the enzymatic activities of these target proteins, evaluated glycolytic stress and mitochondrial respiration by Seahorse tests, and systematically analyzed the changes in metabolites with unbiased LC–MS/MS-based non-targeted metabolomics profiling.

**Results:**

We successfully identified three glycolytic proteins, aldolase A, α-enolase and pyruvate kinase M2 (PKM2), as the binding partners of DA. DA bound to Glu166 of α-enolase, Cys49 and Cys424 of PKM2, and Lys230 of aldolase A, inhibiting the enzymatic activities of α-enolase and PKM2 and thereby impairing ATP synthesis, resulting in mitochondrial dysfunction.

**Conclusions:**

Recent research has revealed that enhancing glycolysis can offer protection against PD. The present study identified that the glycolytic pathway is vulnerable to disruption by DA, suggesting a promising avenue for potential therapeutic interventions. Safeguarding glycolysis against DA-related disruption could be a potential therapeutic intervention for PD.

**Supplementary Information:**

The online version contains supplementary material available at 10.1186/s12964-024-01478-0.

## Introduction

Dopamine (DA) is one of the most abundant neurotransmitters in the nervous system, which plays important roles in physiological and neurological functions [1]. Dopaminergic neurons located in the substantia nigra are the main source of DA [2]. DA deficiency caused by excessive loss of dopaminergic neurons has been demonstrated to strongly correlate with Parkinson’s disease (PD), which is known to be a chronic and progressive neurodegenerative disorder [3, 4]. Although research in PD has made remarkable progress over the past years, the exact mechanism responsible for the progressive degeneration of dopaminergic neurons is still not fully defined.

The biosynthesis, regulation and metabolism of DA have been extensively investigated and nicely summarized in review articles [5–9]. DA is synthesized in the cytosol and then transported to synaptic vesicles, whereas excessive cytosolic DA is toxic [5, 9]. DA toxicity involves modifications of multiple proteins, including mitochondrial proteins [10, 11], lysosomal enzymes [12] as well as PD-implicated proteins [13–15]. Protein-DA modifications could alter protein structure with a consequence of impaired cellular function. Collectively, these studies give a hint that the selective vulnerability of DA neurons in the early stage of PD is associated with the toxicity of DA.

To further investigate the molecular mechanisms of DA-induced toxicity contributing to PD progression, we utilized ABPP [16], a technology in combination with an activity-based probe and chemical proteomics, to comprehensively profile and identify the protein targets of DA in the mouse dopaminergic neuronal MN9D cell line. We found that DA could covalently bind to three glycolytic enzymes, aldolase A, α-enolase and PKM2, resulting in glycolytic and mitochondrial dysfunction. Mitochondrial impairment is considered to be one of the major causes of neurodegenerative diseases including PD [17–19]. Moreover, glycolytic deficits have also been reported to be associated with PD [20]. As enhancing glycolysis is neuroprotective in PD [21], our findings potentially suggest that preventing the modification of glycolytic enzymes by DA could be an alternative treatment strategy for PD.

## Materials and methods

### Regents

Dopamine (DA, purity ≥98%) was obtained from Hubei Wande Chemical Co., Ltd. (Hubei, China). Trypan blue solution, 0.4% was purchased from Thermo Fisher Scientific; Click chemistry reaction reagents including THTPA, TAMRA-azide and biotin-azide, were from Click Chemistry Tools; sodium ascorbate (NaVC) and CuSO_4_ were from Sigma-Aldrich. High capacity neutravidin agarose beads and LC–MS/MS reagents including tetraethylammonium bromide (TEAB), sequencing grade modified trypsin, TMT^10^ plex reagent set and Pierce™ Quantitative Fluorometric Peptide Assay Kit were purchased from Thermo Fisher Scientific; other LC–MS/MS reagents Tris (2-carboxyethyl) phosphine hydrochloride (TECP) and Tris[(1-benzyl-1H-1,2,3-triazol-4-yl)methyl] amine (TBTA) were obtained from Sigma-Aldrich.

The following antibodies were purchased from the Proteintech group: rabbit aldolase A polyclonal antibody (pAb) (#11217–1-AP), mouse GAPDH monoclonal antibody (mAb) (#60004–1-Ig), HRP-conjugated goat anti-mouse (#SA00001–1) and anti-rabbit IgG (#SA00001–2) antibodies. Mouse α-enolase mAb (#sc-100,812) was purchased from Santa Cruz. Rabbit PKM2 pAb (#ab85555) was purchased from Abcam. Alexa Fluor conjugated goat anti-mouse and anti-rabbit IgG antibodies were from Thermo Fisher Scientific.

The following assay kits are commercially available: Enolase activity assay kit (Sigma-Aldrich, #MAK178); Pyruvate kinase activity assay kit (Sigma-Aldrich, #MAK072); ATP assay kit (Beyotime, #S0026); Mitochondrial membrane potential assay kit with JC-1 (Beyotime, #C2006); Annexin V-propidium iodide apoptosis detection kit (KeyGen Biotech, #KGA108); Seahorse XF Cell Mito Stress Test kit (Agilent Technologies, #103015–100); and Seahorse XF Glycolysis Stress Test kit (Agilent Technologies, #103020–100). All assay kits were used according to the manufacturer’s instructions.

### Synthesis of the dopamine-probe (DA-P)

After 4-pentynoic acid (9.8 mg, 0.10 mmol) and HOBT (13.5 mg, 0.10 mmol) were dissolved in 1 ml DCM, EDCI (15.5 mg, 0.10 mmol) and DIPEA (25.8 mg, 0.20 mmol) were added and the mixture was stirred for 1 h to activate the reagent. Dopamine hydrochloride (19.0 mg, 0.10 mmol) and DIPEA (25.8 mg, 0.20 mmol) were added to another flask, and the activated mixture was then dropped into the reaction. The reaction was stirred overnight, quenched by the addition of water, and diluted with DCM. The organic phase was sequentially washed with 1 N HCl, saturated NaHCO_3_ and brine. After the solvent was removed via vacuum evaporation, 14.0 mg dopamine probe was obtained, yielding 60%.

### Cell culture

Mouse MN9D was obtained from Fenghui Biotechnology (Hunan, China). Cells were maintained in MEM plus NEAA (Procell Life Science & Technology) supplemented with 10% fetal bovine serum (FBS) (Thermo Fisher Scientific) at 37 °C in a 5% CO_2_ incubator.

### Striatal neuron cell isolation and culture

Mouse striatum was dissected similarly as described earlier except that neonatal mice (within 24 h after birth) instead of mouse embryos were used [22]. Striatum tissue was minced and digested with trypsin until no large chunks of tissue were observed. The cell suspension was then passed through a 70 μm filter before plating on L-polylysine precoated glass-bottom dishes. After plating, cells were allowed to recover for 1 h at 37 °C in a 5% CO_2_ incubator. Neurobasal-A medium containing 2% B-27 and 1% GlutaMAX was added to maintain the cells. Half of the medium was changed every 48 h. Experiments were carried out on day 8 (DIV8) with well-defined axons and dendrites.

### Dopamine injections in vivo

Animal experimental procedures were performed in accordance with the guidance established by the Care and Use of Laboratory Animals Center of Shenzhen People’s Hospital (Shenzhen, China). C57BL/6 J male mice (19–25 g, 7–8 weeks, Vital River Laboratory Animal Technology, Guangdong, China) were maintained under standard conditions. Mice were first anesthetized with sodium pentobarbital (80 mg/kg). Stereotaxic brain surgery was then used to conduct intrastriatal injection of 2 μl of 0.3 μM dopamine (coordinates from Bregma: A/P + 0.5 mm, L +/− 2.0 mm lateral, D/V − 3.6 mm) over a 25 min period. Mice were killed 24 hours following injection, and the striatal tissue was dissected, weighed and immediately used for ATP detection.

### Fluorescence labeling assay

For the in situ fluorescence labeling assay, samples were treated as previously described [23–25]. Briefly, MN9D cells were incubated with or without the DA-probe (0, 12.5, 25, 50 and 100 μM) for 3 h at 37 °C. For DA competition experiments, cells were pre-incubated with indicated concentrations of DA for 4 h, followed by 100 μM DA-P treatment for 3 h. After washing cells with ice-cold PBS, cells were lysed in RIPA buffer and cleared by centrifugation at 12,000 rpm for 15 min. Next, equal amounts of lysate samples from each group were incubated with the premixed click reaction cocktail (1 mM NaVc, 100 mM THPTA, 1 mM CuSO_4_ and 50 mM TAMRA-N_3_) at room temperature for 2 h. Prechilled acetone was then added to precipitate the proteins. Proteins were then dissolved by sonication. An equal volume of SDS–PAGE sample buffer was added to each protein sample. Proteins were finally resolved by 10% SDS-PAGE. Gels were scanned using the Sapphire Biomolecular Imager (Azure Biosystem), and image analysis was performed with ImageJ.

For fluorescence labeling of recombinant protein, purified proteins were incubated with DA-probe and shaken at room temperature for 1 h. For competition experiments, competitors DA or IAA were added to the concentrations as indicated for 1 h following with another 1 h of probe incubation. Probe-labeled proteins were ligated to TAMRA-azide by copper-catalyzed azide–alkyne cycloaddition reaction (CuAAC) and separated in SDS–PAGE gel. Samples were visualized by fluorescence scanning as described above.

### ABPP-based protein target identification

MN9D cells cultured in 10 cm dishes were treated with DA-P (100 μM) for 3 h. For the competition group, cells were first incubated with the competitor DA (800 μM) for 4 h before treatment with DA-P. After incubation, cells were lysed and conjugated with biotin-azide by click chemistry reaction. Clicked proteins were then precipitated with ice-cold acetone and air-dried. Next, pellets were dissolved in 1.5% SDS in PBS. The dissolved protein samples were further diluted with PBS to a final concentration of 0.1% SDS. The resulting solution was subjected to incubation with 50 μl of pre-washed streptavidin beads for 4 h at room temperature. Subsequently, the beads were washed thrice with 1% SDS, 0.1% SDS, 6 M urea and PBS.

Proteins enriched on the beads were then reduced and alkylated by dithiothreitol (DTT) and iodoacetamide (IAA) respectively. Next, trypsin was added, and the samples were incubated at 37 °C overnight. Following digestion, peptides were desalted on a C18 column and labeled with TMT^10^ plex Mass Tag Labeling reagents. Finally, the samples were analyzed by LC–MS/MS (Thermo Fisher).

### Immunofluorescence labeling

MN9D cells grown on Φ 12 mm glass coverslips were treated with DA-P in the absence or presence of competitor DA. After 3 h of incubation, the cells were fixed with 4% paraformaldehyde in PBS at room temperature for 20 min before permeabilization with 0.2% Triton X-100. The cells were then subjected to a click reaction for 2 h at room temperature. After washing with PBS, the coverslips were mounted in Mowiol 4–88. For colocalization experiments, after the click reaction, cells were incubated with diluted primary antibody followed by diluted fluorescence-conjugated secondary antibody. All images were acquired with fluorescence microscopy.

### Cellular thermal shift assay (CETSA)

CETSA was performed according to previously described procedures [26, 27]. Briefly, MN9D protein lysates were incubated with DA for 2 h at room temperature. After incubation, cell lysates were divided into 10 equal parts and heated at the designated temperatures for 3 min. Heated samples were quickly cooled on ice followed by centrifugation at 20,000 x g for 20 min at 4 degrees. Western blotting was subsequently conducted to detect soluble proteins.

### Purification of his-tagged aldolase a, α-enolase and PKM2 proteins

pCold II, encoding His-tagged human aldolase A, α-enolase or PKM2, was transformed into BL21 *E. coli* cells. After induction by 0.3 mM isopropyl β-D-1-thiogalactopyranoside (IPTG), bacterial pellets were lysed by sonication in lysis buffer (20 mM Tris-HCl pH 8.0, 200 mM NaCl, 1 mM PMSF and 1× cOmplete™ Protease Inhibitor Cocktail). After high-speed centrifugation, the supernatant was collected and incubated with pre-washed Ni-NTA agarose beads for 2 h at 4 degrees. After extensive washing using a buffer containing 20 mM Tris-HCl pH 8.0, 200 mM NaCl and 25 mM imidazole, bead-immobilized His-tagged aldolase A, α-enolase and PKM2 were eluted with elution buffer containing 250 mM imidazole.

### Metabolomic analysis by LC–MS/MS

MN9D cells incubated with 400 μM DA for 12 h were collected. Metabolites were extracted from the cell samples following the published protocol [28] for LC–MS/MS analysis. Raw data were imported into Compound Discoverer 3.1 software for peak alignment and peak picking. Each metabolite was quantitated by the mzCloud, mzVault and MassList databases. MetaboAnalyst 5.0 web servers and statistical software R (R version R-4.1.1) were applied to perform the metabolite statistical analysis. Student’s *t*-test and fold change (FC) were used to identify differential features. A volcano plot was produced with GraphPad Prism to highlight the significant differentially abundant metabolites between the DA treatment and control groups. KEGG pathway enrichment analysis was performed on differentially abundant metabolites.

### Molecular docking

The 3D structure SDF file of DA was obtained from PubChem to generate the PDBQT file by Open Babel [29]. Structures of human aldolase A (PDB entry 6XMH) and PKM2 (PDB entry 4B2D) were downloaded from RCSB PDB. As the structure of the open active state of human α-enolase is not available, we performed homology modeling using yeast α-enolase (PDB entry 1L8P, open active state) as a template [30]. After that, these receptor structure files were transferred into PDBQT format through the AutoDock tool (ADT) [31]. Docking analysis was performed using AutoDock Vina (v1.2.3) [32]. The grid box with a side length parameter of 30 was centered on the enzymatic active sites. In addition, the exhaustiveness parameter was set to 32 to find a lower affinity binding pose. The PLIP web tool [33] was used to analyze the docking results, which were eventually visualized in PyMOL.

### Statistics

Data were analyzed using GraphPad Prism 9.0. One-way ANOVA followed by Turkey’s test was used to evaluate statistical significance in multiple groups, and Student’s *t* test was used to compare two groups. *p* < 0.05 was considered statistically significant.

## Results

### Design, synthesis and characterization of dopamine probe

**Fig. 1:**
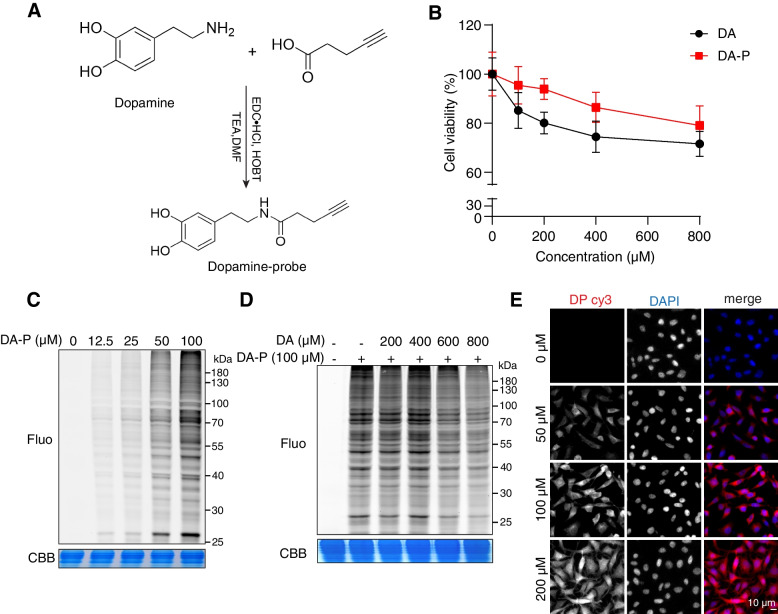
Dopamine probe (DA-P) serves as an effective chemical tool for protein labeling in MN9D cells. **A** Schematic illustration of the synthesis route for DA-P. Structures of DA and DA-P are shown. **B** Trypan blue exclusion test of MN9D cell viability following treatment with different concentrations of DA or DA-P (0, 100, 200, 400 and 800 μM) for 24 h. **C** In-gel fluorescence visualization of proteins labeled by indicated concentrations of DA-P for 3 h in live MN9D cells through the copper-catalyzed azide–alkyne cycloaddition reaction (CuAAC). **D** In-gel fluorescence visualization of MN9D proteome after pre-treatment with indicated concentrations of DA for 4 h followed by 100 μM DA-P labeling. **E** Representative fluorescence images of MN9D cells following treatment with 0 μM, 50 μM, 100 μM or 200 μM of DA-P, respectively for 3 h. Cells were processed for CuAAC click reaction with TAMRA-azide. Error bars, mean ± SEM. Scale bar, 10 μm. Fluo fluorescence, CBB coomassie brilliant blue

To identify the potential protein targets of dopamine in living systems, we developed a dopamine probe (DA-P) containing a clickable alkyne group at the amino terminus of dopamine (Fig. S1). The detailed synthesis process of DA-P is presented in Fig. 1A. We then evaluated and compared the cytotoxic effects of DA and DA-P in undifferentiated dopaminergic MN9D cells. As shown in Fig. 1B, both DA and DA-P exhibited similar dose-related toxicity profiles. Next, MN9D cells were incubated in situ with DA-P for 3 h and lysed, followed by the attachment of TAMRA-azide by copper-catalyzed azide–alkyne cycloaddition reaction (CuAAC). As observed via SDS-PAGE and in-gel fluorescence scanning, DA-P could label proteins effectively in a concentration-dependent manner (Fig. 1C). A 100 μM concentration of DA-P was selected for the subsequent assay as it could yield an optimal labeling intensity. In the competition experiment, we found that the labeling of DA-P could be partially outcompeted by excess DA (Fig. 1D), indicating that the binding of DA-P is specific. Subcellular localization of DA-P was also visualized by click chemistry reaction, which was found to distribute throughout the cytoplasm in MN9D cells (Fig. 1E).

### DA directly binds to three glycolytic enzymes: aldolase a, α-enolase and PKM2

**Fig. 2:**
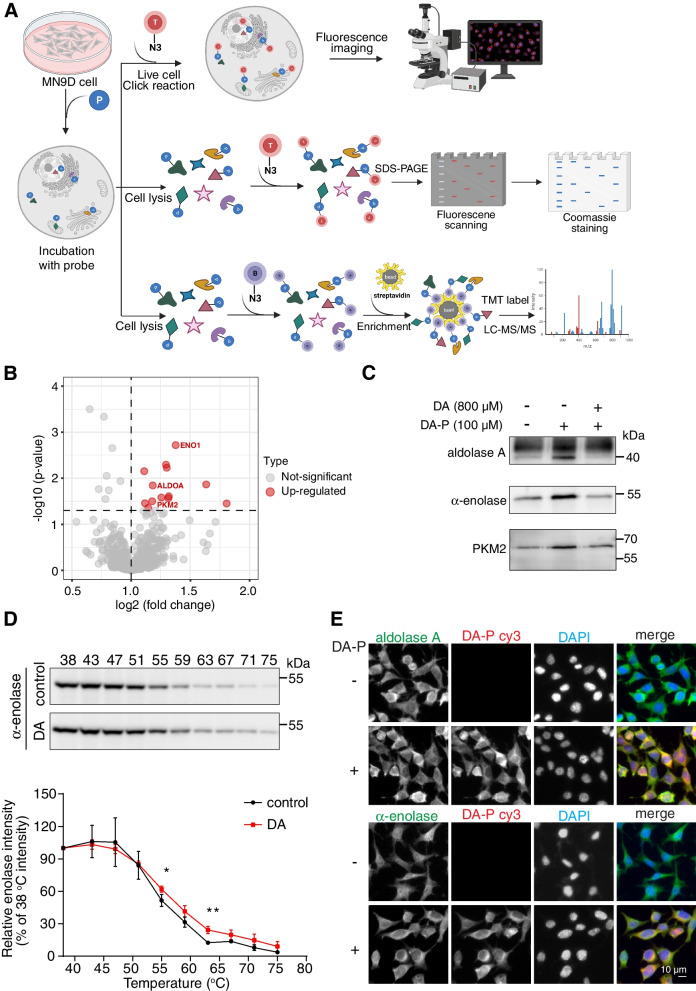
Identification of aldolase A, α-enolase and PKM2 as the binding partners of DA in MN9D cells. **A** ABPP workflow for the identification of the DA protein targets. Live cells were incubated with DA-probe (blue circle). For fluorescence imaging, cells were fixed and permeabilized, followed by the attachment of TAMRA-azide tag (red circle) by CuAAC reaction; otherwise, cells were lysed and the DA-P labeled proteome were ligated by CuAAC to TAMRA-azide for fluorescence SDS-PAGE analysis, or to biotin azide (purple circle) for LC–MS/MS based protein targets identification. **B** Volcano plot representing proteins labeled by DA-P (100 μM) for 3 h in live cells versus samples pre-treated with 800 μM DA for 4 h followed by co-treatment with DA-P (100 μM) for 3 h (fold change ≥ 2 and *p* value < 0.05). Red: upregulated genes. **C** In situ pull-down to verify the interaction between DA and aldolase A, α-enolase and PKM2 proteins. Cells treated with 100 μM DA-P or with an excess of the DA (800 μM, 4 h) plus the DA-P for 3 h were lysed, followed by the attachment of biotin-azide by CuAAC reaction. **D** CETSA to verify DA binding to α-enolase. Cell lysates incubated with 100 μM DA for 2 h were subjected to CESTA-western blot analysis. **E** Representative fluorescence images of intracellular aldolase A or α-enolase in MN9D cells incubated with 100 μM DA-P for 3 h. Error bars, mean ± s.d. Scale bar, 10 μm. *, *p* < 0.05; **, *p* < 0.01

Having characterized the DA-probe in living cells, we proceeded to explore the direct binding partners of DA by the ABPP approach. As illustrated in Fig. 2A, DA-P labeled protein lysates were reacted with biotin-alkyne, enriched on streptavidin beads, separated by SDS-PAGE, and identified using LC–MS/MS. We generated a volcano plot to visualize the significance and magnitude of binding changes in proteins between the DA-P group and the DA competition group (Fig. 2B). Meanwhile, proteins with fold change ≥ 2 and *p* value < 0.05 were depicted as potential targets of DA (Fig. 2B, highlighted in red). We noticed three glycolytic enzymes in the list: aldolase A, α-enolase and PKM2, which act in the fourth step, the second to last step and the final step in glycolysis, respectively. To further confirm the direct interactions between DA and the identified glycolytic enzymes, we performed a pull-down assay followed by immunoblotting in DA-P treated MN9D cells. As expected, we found that all three glycolytic enzymes were pulled down by DA-P. In particular, an 8-fold excess of DA added to the cells 4 h before probe incubation was able to efficiently block the protein binding to DA-P (Fig. 2C). We also conducted a cellular thermal shift assay (CETSA) to evaluate the binding efficiency between target glycolytic proteins and DA. CETSA has been used extensively in recent years to detect the interactions between ligands and proteins, which is based on the principle of altered protein thermal stabilization or destabilization upon ligand binding [34–36]. We then compared and measured the amounts of soluble α-enolase, PKM2 or aldolase A remaining in cells after heating at a panel of temperatures in the absence or presence of DA. Interestingly, the thermal stability of α-enolase was significantly enhanced in the presence of DA (Fig. 2D), whereas PKM2 and aldolase A showed some reduction in thermal stability after DA treatment, suggesting that DA binding could destabilize PKM2 and aldolase A protein (Fig. S2A&B). In addition, colocalization of DA-P with aldolase A, α-enolase and PKM2 in MN9D cells was also observed (Fig. 2E, Fig. S2C). These findings suggested that DA could directly bind to aldolase A, α-enolase and PKM2 in cultured MN9D cells.

### DA inhibits the enzymatic activities of α-enolase and PKM2

**Fig. 3:**
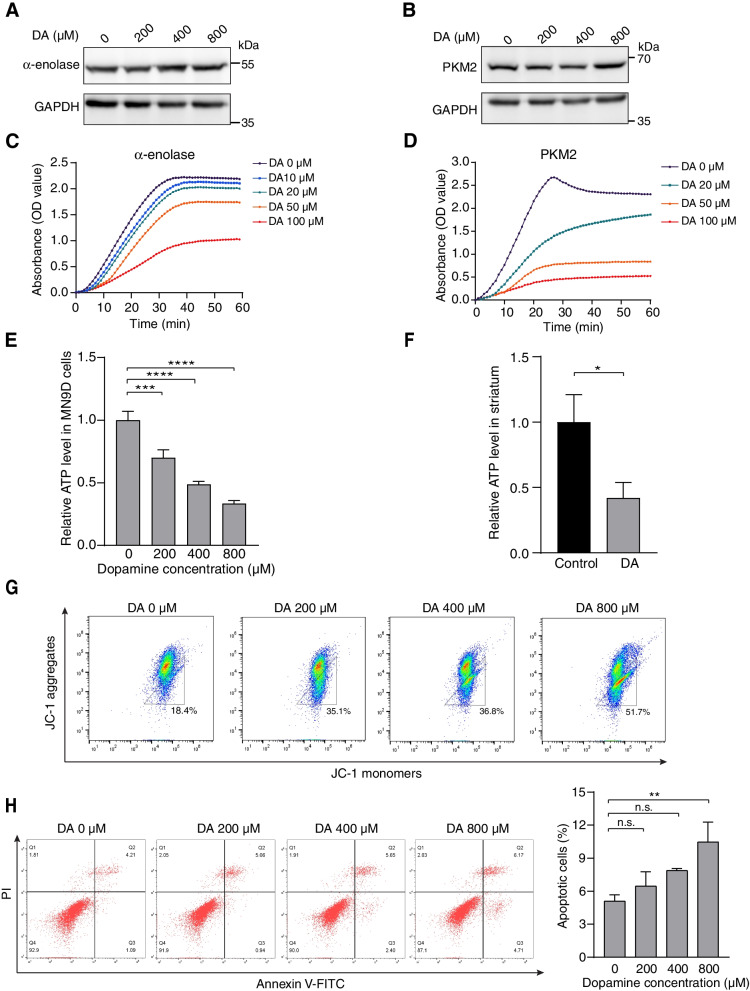
DA suppresses the enzymatic activities of α-enolase and PKM2. **A**, **B** Immuno-blots showing the levels of endogenous α-enolase and PKM2 after incubation with indicated concentrations of DA for 24 h. **C**, **D** Measurement of the catalytic activities of recombinant α-enolase and PKM2 in the presence of indicated concentrations of DA treatment. **E** Quantification of relative ATP levels in MN9D cells treated with 0 μM, 200 μM, 400 μM or 800 μM of DA for 24 h. **F** Measurement of ATP levels in the DA-injected mouse striatum. ATP were measured one day following an intrastriatal injection of DA (0.3 μM). **G** Flow cytometric analysis of JC-1 aggregates in MN9D cells treated with DA for 24 h. **H** Representative flow cytometric plots and quantification results of apoptotic cells after 24 h of DA exposure. Error bar, mean ± s.d.;. n.s., not significant; *, *p* < 0.05; **, *p* < 0.01; ***, *p* < 0.001; ****, *p* < 0.0001

Our next objective was to test whether DA can affect the catalytic activities of identified glycolytic enzymes. Although the expression levels of aldolase A, α-enolase and PKM2 were barely affected by DA (Fig. 3A&B, Fig. S3A), the enzymatic activities of recombinant α-enolase and PKM2 were significantly reduced by incubation with DA (Fig. 3C&D). In the mammalian central nervous system, DA is produced by the dopaminergic neurons of the midbrain and released in the striatum. Hence, we further tested whether DA affects the catalytic activity of α-enolase in striatum neurons and mouse midbrain tissue. Striatal neurons in culture for 8 days were lysed and mouse midbrain tissue was homogenized, incubated with 0 μM, 50 μM, 100 μM and 200 μM of DA and subjected to the measurement of α-enolase enzymatic activity. We observed that treatment with DA resulted in similar reduction levels of α-enolase enzymatic activity in both striatal neuron lysates and mouse midbrain tissue. Moreover, the inhibition of the α-enolase catalytic activity in both lysates is DA concentration-dependent (Fig. S3B&C). Glycolysis is one of the first metabolic pathways to produce ATP, and PKM2 functions in the last glycolytic step, which converts phosphoenopyruvate to pyruvate with the concomitant production of ATP [37]. Thus, we hypothesized that ATP production is also reduced when the activities of glycolytic enzymes are impaired. Indeed, a dose-dependent decrease in intracellular ATP following pretreatment with increasing concentrations of DA for 24 h was observed (Fig. 3E). Accordingly, we also examined the effect of DA overdose on energy generation in vivo. ATP was measured in the mouse striatum 24 h following an intrastriatal injection of 0.3 μM DA. As shown in Fig. 3F, DA significantly decreased ATP levels in the striatum compared with the control group. We then proceeded to test whether mitochondrial function is also impaired after DA exposure. The mitochondrial membrane potential (ΔΨm), one of the most reliable indicators of mitochondrial function, was evaluated by flow cytometry analysis using JC-1 fluorescent probes. Following the administration of DA, the percentage of cells with green-fluorescent JC-1 monomers increased from 19.6% in the control group to 53.3% in the 800 μM DA treatment group, suggesting the earliest event in mitochondrial dysfunction (Fig. 3G). Lastly, the effect of DA on cell apoptosis was also explored. The percentage of apoptotic cells was significantly increased by DA treatment, although it did not cause a high level of apoptosis (Fig. 3H). These results implied that the enzymatic activities of α-enolase and PKM2 are compromised upon DA exposure, which might further induce mitochondrial damage.

### DA binds to Glu166 in α-enolase

**Fig. 4:**
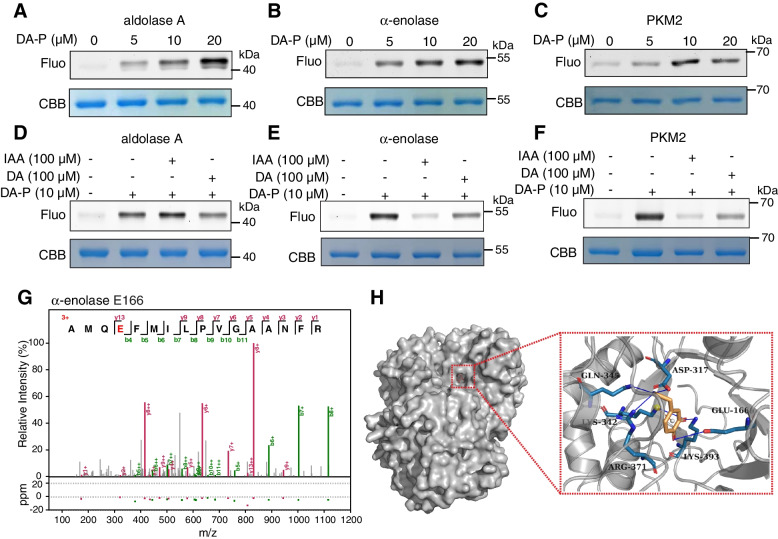
DA could bind to E166 residue of α-enolase**. A**-**C** In-gel fluorescence visualization of recombinant human aldolase A, α-enolase or PKM2 after labeling with different concentrations of DA-P. **D**-**F** In-gel fluorescence visualization of recombinant human aldolase A, α-enolase and PKM2 labeled by DA-P in the presence or absence of the competitors DA or IAA. **G** MS/MS spectrum of human α-enolase treated with 100 μM DA for 60 min. The amino acid E highlighted in red represents the glutamic acid bound to DA. **H** A binding model of DA with human α-enolase predicted by molecular docking. Fluo, fluorescence; CBB, coomassie brilliant blue

We next sought to investigate how DA interacts with these three glycolytic enzymes. We first incubated recombinant human aldolase A, α-enolase and PKM2 with different concentrations of DA-P (0–20 μM) followed by the attachment of TAMRA-azide by CuAAC. The labeling intensities of these proteins by DA-P gradually increased with increasing concentrations of DA-P (Fig. 4A-C). Furthermore, 100 μM DA could efficiently compete with DA-P for binding to these three proteins (Fig. 4D-F). Meanwhile, we noticed that iodoacetamide (IAA), a general cysteine alkylating agent, was able to compete away the labeling of α-enolase and PKM2 but not aldolase A by DA-P, suggesting that some reactive cysteine residues in α-enolase and PKM2 might be the binding sites of DA (Fig. 4D-F, Fig. S4A-C**)**. Indeed, using tandem mass spectrometry, we identified that Cys49 and Cys424 (a site that can affect the subunit interaction and enzymatic activity of PKM2 [38]) residues in PKM2 were modified by DA (Fig. S4F-H). Interestingly, Glu166, an active-site residue responsible for 2-phosphoglycerate binding to α-enolase [39], was detected as the binding site of DA in α-enolase (Fig. 4G). The molecular docking analysis also coincided with the recognition that DA could interact with Glu166 in α-enolase (Fig. 4H). For aldolase A, we speculated that Lys230 (the active site residue) [40] might be the modified site of DA (Fig. S4D&E).

### Metabolic dysfunction caused by DA via inducing mitochondrial damage

**Fig. 5:**
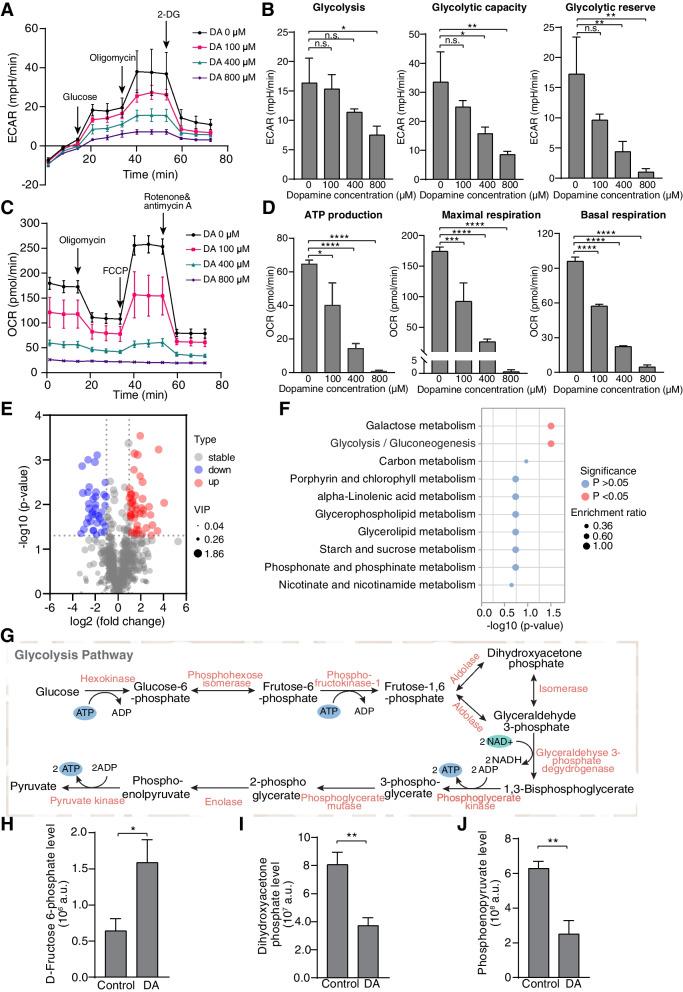
Metabolic dysfunction caused by DA-induced mitochondrial damage. **A**, **B **Measurement of the extracellular acidification rate (ECAR) and its indicators in MN9D cells treated with indicated concentrations of DA for 12 h. **C**, **D** Measurement of the oxygen consumption rate (OCR) and its indicators in MN9D cells treated with indicated concentrations of DA for 12 h. **E** Volcano plot of the different metabolites in MN9D cells treated with 400 μM DA. Red and blue dots represent the upregulated and downregulated metabolites respectively. **F** KEGG pathway enrichment analysis of DA-targeted metabolites. Top 10 pathways are shown. **G** Schematic representation of the glycolysis pathway. **H**-**J** Quantification of glycolysis intermediates after 400 μM DA treatment for 12 h. All error bars represent the mean ± s.d. n.s., not significant; *, *p* < 0.05; **, *p* < 0.01; ***, *p* < 0.001; ****, *p* < 0.0001

As the powerhouse of the cell, mitochondria play a central role in cellular metabolism and homeostasis [41]. Mitochondrial dysfunction has been implicated in various neurodegenerative diseases, including PD [42]. Our results on protein species modified by DA might provide a link between mitochondrial dysfunction and PD pathogenesis. Thus, we further investigated the impact of DA on mitochondrial functions. Cellular glycolysis was first examined using the Seahorse glycolytic stress test, which revealed that glycolysis (measured as extracellular acidification rate, ECAR), glycolytic capacity and the glycolytic reserve in MN9D cells decreased remarkably in a dose-dependent manner by 12 h of DA treatment (Fig. 5A&B). Similarly, we evaluated mitochondrial respiration by conducting the Seahorse cell mitochondrial stress test. A significant reduction in oxygen consumption rate (OCR) was observed after DA exposure (Fig. 5C). ATP production, basal respiration, maximal respiration, proton leakage and spare respiration rate, which were all significantly lower in the DA treatment group than in the control group (Fig. 5D, Fig. S5A-C). Collectively, these findings provided strong evidence that cells are incapable of responding to an energetic demand after DA exposure, since excessive DA impairs key glycolytic enzymes and thus reduces mitochondrial ATP production.

Mitochondria are central to cellular metabolism. To further characterize the impairment of metabolic processes after DA exposure, LC–MS/MS-based-nontargeted metabolomics profiling was performed. By metabolomics analysis, the differentially abundant metabolites in control and 400 μM of DA-treated samples were screened and identified (Fig. S5D). The dysregulated metabolites were visualized in a volcano plot (Fig. 5E). In addition, KEGG enrichment annotation analysis was conducted to annotate the unique significant metabolite pathways affected by DA, which were found to be galactose metabolism and glycolysis/gluconeogenesis (Fig. 5F, Fig. S5E). On the basis of our above LC–MS/MS results (Fig. 2B), we chose to focus on the glycolysis pathway (Fig. 5G). DA treatment significantly increased the level of D-fructose 6-phosphate, an upstream substrate of aldolase A (Fig. 5H), and decreased the level of the aldolase A downstream product dihydroxyacetone phosphate (Fig. 5I). Likewise, a statistically significant reduction was also observed in the level of the α-enolase catalytic product phosphoenopyruvate (Fig. 5J). Overall, these data demonstrated that exposure to DA could result in aldolase A and enolase inactivation, which further impair key metabolic pathways in glycolysis. This is consistent with our ABPP results mentioned above.

## Discussion

In this study, we designed and synthesized a DA-probe that mimics endogenous DA to perform an ABPP assay in the MN9D cell line. We identified three glycolytic enzymes, aldolase A, α-enolase and PKM2, conjugated to DA. Covalent modifications of these proteins by DA inhibited enzymatic functions and impaired ATP synthesis, resulting in mitochondrial dysfunction.

Glycolysis is the metabolic process in which glucose is converted to pyruvate with the subsequent generation of ATP [43]. The mammalian brain depends mostly on glucose as a source of energy [44]. Changes in glycolysis are observed in several neurodegenerative diseases [21, 43, 45]. Indeed, phosphoglycerate kinase 1 (PGK-1), the first ATP-producing enzyme in glycolysis, has been linked to PD [46], as its deficiency was generally found in male PD patients [47]. Enhancement of PGK-1 activity by the alpha 1 receptor antagonist terazosin [48] could attenuate neurodegeneration in genetic models of PD [49]. Despite PGK-1, the functions of other glycolytic enzymes in PD are not fully clarified. PKM2 is another ATP-generating enzyme in the glycolytic pathway that catalyzes the conversion of phosphoenolpyruvate and ADP to pyruvate and ATP. Recently, several studies [50, 51] have indicated that PKM2 might act as a neuroprotector against oxidative stress by promoting GSH biosynthesis. In addition, Jin et al. reported the association of aldolase A with the PD-linked proteins α-synuclein and DJ-1 using a SILAC in dopaminergic MES cells [52]. α-enolase, together with carbonic anhydrase 2 (Car2) and lactate dehydrogenase 2 (Ldh2), was found to be oxidatively inactivated in a mouse model of PD [53]. Therefore, such findings provide a new treatment option for PD, namely, treatment through glycolysis.

In this frame, our results are particularly interesting. Our analysis identified PKM2, aldolase A and α-enolase, which are all glycolytic enzymes involved in glycolysis. Although aldolase A has been previously reported as a DA-conjugated protein, researchers only analyzed the abundance change of the protein [54]. Our study revealed that DA bound to these three enzymes blocks their catalytic activity, thereby disrupting glycolysis. Moreover, the specific binding sites of DA to these three proteins were tested. Disruption of the glycolytic pathway results in decreased ATP production. The effects of DA exposure on glycolysis were further validated by the Seahorse glycolytic stress test and metabolomics analysis. Defects in mitochondrial respiration were also observed after DA treatment. DA could lower the mitochondrial membrane potential and reduce the mitochondrial respiration chain.

To date, the mainstay treatment for PD is still the administration of the DA precursor levodopa [55]. However, long-term exposure to levodopa frequently leads to dyskinesia as well as cognitive impairment, suggesting that DA is a double-edged sword, and that increasing the DA concentration could damage neuronal cells [56]. Thus, the identification of protein targets of DA and its metabolites is critical to uncover the relationship between DA and neurodegenerative diseases. The discovery of three glycolytic proteins, aldolase A, α-enolase and PKM2, as DA binding partners in our study highlights the importance of energy metabolism in PD. Overall, our findings lead to the hypothesis that protecting the glycolysis pathway from DA disruption represents a promising new direction of therapy for PD.

## Conclusions

In summary, we systemically studied the toxicity of DA by utilizing ABPP technology in combination with metabolomics analysis. We identified three glycolytic proteins, aldolase A, α-enolase and PKM2, as the binding partners of DA. Disruption of glycolysis by DA exposure impaired mitochondrial functions. Glycolysis has been recently found to have a neuroprotective role in PD. Thus, our findings further indicate that protecting the glycolysis pathway from DA disruption could serve as a novel approach to mitigating the progression of PD.

### Supplementary Information

**Additional file 1: Fig. S1.** ^1^H NMR and ^13^C NMR of dopamine probe (DA-P). **Fig. S2. **CESTA and immunofluorescence to verify DA binding to PKM2 and aldolase A. A&B CETSA to verify DA binding to PKM2 or aldolase A. Cell lysates incubated with 100 µM DA for 2 h were subjected to CESTA-western blot analysis. C Representative fluorescence images of intracellular PKM2 in MN9D cells following labeling with 100 µM DA-P for 3 h. Error bars, mean ± s.d. Scale bar, 10 µm. *, *p* < 0.05. **Fig. S3.** DA suppresses the enzymatic activity of neuron α-enolase. A The immuno-blots showing the level of endogenous aldolase A after incubation with indicated concentrations of DA for 24 h. B Measurement of the catalytic activity of α-enolase extracted from cultured striatal neurons with the treatment of indicated concentrations of DA. C Measurement of the catalytic activity of α-enolase extracted from mouse midbrain tissue with the treatment of indicated concentrations of DA. **Fig. S4. **DA could bind to K230 residue of aldolase A, and Cys49 and Cys424 residues of PKM2. A-C In-gel fluorescence visualization of recombinant human aldolase A, α-enolase and PKM2 labeled by IAA-yne in the presence or absence of the competitors DA or IAA. D MS/MS spectrum of human aldolase A treated with 100 µM DA for 60 min. E A binding model of DA with human aldolase A predicted by molecular docking. F&G MS/MS spectrum of human PKM2 treated with 100 µM DA for 60 min. H A binding model of DA with human PKM2 predicted by molecular docking. **Fig. S5. **Metabolic dysfunction caused by DA via inducing mitochondrial damage. A-C Measurement of indicators of OCR rate in MN9D cells under different concentrations of DA (0, 100, 400 and 800 µM) treatment for 12 h. D Heatmap showing top up and down metabolites after 400 µM DA incubation for 12 h. E KEGG pathway enrichment analysis of DA-targeted metabolites. Top 25 pathways are shown. All error bars represent mean ± s.d. n.s. not significant; *, *p* < 0.05; **, *p* < 0.01; ***, *p* < 0.001; ****,* p* < 0.0001.

## Data Availability

The datasets used and/or analyzed during the current study are available from the corresponding author on reasonable request.

## Abbreviations

PD: Parkinson’s disease
DA: dopamine
DA-P: dopamine-probe
ABPP: activity-based protein profiling
CuAAC: copper-catalyzed azide–alkyne cycloaddition reaction
LC–MS/MS: liquid chromatography-tandem mass spectrometry
Fluo: fluorescence
CBB: Coomassie brilliant blue
CESTA: cellular thermal shift assay
PKM2: pyruvate kinase M2
IAA: iodoacetamide
ECAR: extracellular acidification rate
OCR: oxygen consumption rate

## References

[1] Ko JH, Strafella AP (2012). Dopaminergic neurotransmission in the human brain: new lessons from perturbation and imaging. Neuroscient..

[2] Björklund A, Dunnett SB (2007). Dopamine neuron systems in the brain: an update. Trends Neurosci..

[3] Barzilai A, Melamed E, Shirvan A (2001). Is there a rationale for neuroprotection against dopamine toxicity in Parkinson's disease?. Cell Mol Neurobiol..

[4] Surmeier DJ, Obeso JA, Halliday GM (2017). Selective neuronal vulnerability in Parkinson disease. Nat Rev Neurosci..

[5] Meiser J, Weindl D, Hiller K (2013). Complexity of dopamine metabolism. Cell Commun Signal..

[6] Monzani E, Nicolis S, Dell'Acqua S, Capucciati A, Bacchella C, Zucca FA, Mosharov EV, Sulzer D, Zecca L, Casella L (2019). Dopamine, oxidative stress and protein-Quinone modifications in Parkinson's and other neurodegenerative diseases. Angew Chem Int Ed Engl..

[7] Eisenhofer G, Kopin IJ, Goldstein DS (2004). Catecholamine metabolism: a contemporary view with implications for physiology and medicine. Pharmacol Rev..

[8] Liu C, Kaeser PS (2019). Mechanisms and regulation of dopamine release. Curr Opin Neurobiol..

[9] Latif S, Jahangeer M, Maknoon Razia D, Ashiq M, Ghaffar A, Akram M, El Allam A, Bouyahya A, Garipova L, Ali Shariati M, Thiruvengadam M, Azam Ansari M (2021). Dopamine in Parkinson's disease. Clin Chim Acta..

[10] Hauser DN, Dukes AA, Mortimer AD, Hastings TG (2013). Dopamine quinone modifies and decreases the abundance of the mitochondrial selenoprotein glutathione peroxidase 4. Free Radic Biol Med..

[11] Belluzzi E, Bisaglia M, Lazzarini E, Tabares LC, Beltramini M, Bubacco L (2012). Human SOD2 modification by dopamine quinones affects enzymatic activity by promoting its aggregation: possible implications for Parkinson's disease. PLoS One..

[12] Burbulla LF, Song P, Mazzulli JR, Zampese E, Wong YC, Jeon S, Santos DP, Blanz J, Obermaier CD, Strojny C, Savas JN, Kiskinis E, Zhuang X, Krüger R, Surmeier DJ, Krainc D (2017). Dopamine oxidation mediates mitochondrial and lysosomal dysfunction in Parkinson's disease. Science..

[13] Girotto S, Sturlese M, Bellanda M, Tessari I, Cappellini R, Bisaglia M, Bubacco L, Mammi S (2012). Dopamine-derived quinones affect the structure of the redox sensor DJ-1 through modifications at Cys-106 and Cys-53. J Biol Chem..

[14] Mor DE, Tsika E, Mazzulli JR, Gould NS, Kim H, Daniels MJ, Doshi S, Gupta P, Grossman JL, Tan VX, Kalb RG, Caldwell KA, Caldwell GA, Wolfe JH, Ischiropoulos H (2017). Dopamine induces soluble α-synuclein oligomers and nigrostriatal degeneration. Nat Neurosci..

[15] Cappai R, Leck SL, Tew DJ, Williamson NA, Smith DP, Galatis D, Sharples RA, Curtain CC, Ali FE, Cherny RA, Culvenor JG, Bottomley SP, Masters CL, Barnham KJ, Hill AF (2005). Dopamine promotes alpha-synuclein aggregation into SDS-resistant soluble oligomers via a distinct folding pathway. FASEB J..

[16] Chen X, Wang Y, Ma N, Tian J, Shao Y, Zhu B, Wong YK, Liang Z, Zou C, Wang J (2020). Target identification of natural medicine with chemical proteomics approach: probe synthesis, target fishing and protein identification. Signal Transduct Target Therapy..

[17] Berman SB, Hastings TG (1999). Dopamine oxidation alters mitochondrial respiration and induces permeability transition in brain mitochondria: implications for Parkinson's disease. J Neurochem..

[18] Bisaglia M, Soriano ME, Arduini I, Mammi S, Bubacco L (2010). Molecular characterization of dopamine-derived quinones reactivity toward NADH and glutathione: implications for mitochondrial dysfunction in Parkinson disease. Biochim Biophys Acta..

[19] Biosa A, Arduini I, Soriano ME, Giorgio V, Bernardi P, Bisaglia M, Bubacco L (2018). Dopamine oxidation products as mitochondrial endotoxins, a potential molecular mechanism for preferential neurodegeneration in Parkinson's disease. ACS Chem Neurosci..

[20] Zhang X, Alshakhshir N, Zhao L (2021). Glycolytic metabolism Brain Resilience, and Alzheimer's Disease. Front Neurosci..

[21] Naeem U, Arshad AR, Jawed A, Eqbal F, Imran L, Khan Z, Ijaz F (2022). Glycolysis: the next big breakthrough in Parkinson's disease. Neurotox Res..

[22] Schmidt ER, Morello F, Pasterkamp RJ. Dissection and culture of mouse dopaminergic and striatal explants in three-dimensional collagen matrix assays. J Vis Exp. 2012;61 10.3791/3691 PMC3460578 22473326

[23] Wang J, Zhang CJ, Chia WN, Loh CC, Li Z, Lee YM, He Y, Yuan LX, Lim TK, Liu M, Liew CX, Lee YQ, Zhang J, Lu N, Lim CT, Hua ZC, Liu B, Shen HM, Tan KS, Lin Q (2015). Haem-activated promiscuous targeting of artemisinin in plasmodium falciparum. Nat Commun..

[24] Liu DD, Luo P, Gu L, Zhang Q, Gao P, Zhu Y, Chen X, Guo Q, Zhang J, Ma N, Wang J (2021). Celastrol exerts a neuroprotective effect by directly binding to HMGB1 protein in cerebral ischemia-reperfusion. J Neuroinflammation..

[25] Zhang Q, Luo P, Xia F, Tang H, Chen J, Zhang J, Liu D, Zhu Y, Liu Y, Gu L, Zheng L, Li Z, Yang F, Dai L, Liao F, Xu C, Wang J (2022). Capsaicin ameliorates inflammation in a TRPV1-independent mechanism by inhibiting PKM2-LDHA-mediated Warburg effect in sepsis. Cell Chem Biol..

[26] Dai L, Zhao T, Bisteau X, Sun W, Prabhu N, Lim YT, Sobota RM, Kaldis P, Nordlund P (2018). Modulation of protein-interaction states through the cell cycle. Cell..

[27] Jafari R, Almqvist H, Axelsson H, Ignatushchenko M, Lundbäck T, Nordlund P, Martinez Molina D (2014). The cellular thermal shift assay for evaluating drug target interactions in cells. Nat Protoc..

[28] Souza AL, Patti GJ (2021). A protocol for untargeted Metabolomic analysis: from sample preparation to data processing. Methods Mol Biol..

[29] O'Boyle NM, Banck M, James CA, Morley C, Vandermeersch T, Hutchison GR (2011). Open babel: an open chemical toolbox. J Cheminform..

[30] Poyner RR, Larsen TM, Wong SW, Reed GH (2002). Functional and structural changes due to a serine to alanine mutation in the active-site flap of enolase. Arch Biochem Biophys..

[31] Morris GM, Huey R, Lindstrom W, Sanner MF, Belew RK, Goodsell DS, Olson AJ (2009). AutoDock4 and AutoDockTools4: automated docking with selective receptor flexibility. J Comput Chem..

[32] Eberhardt J, Santos-Martins D, Tillack AF, Forli S (2021). AutoDock Vina 1.2.0: new docking methods, expanded force field, and Python bindings. J Chem Inf Model..

[33] Adasme MF, Linnemann KL, Bolz SN, Kaiser F, Salentin S, Haupt VJ, Schroeder M (2021). PLIP 2021: expanding the scope of the protein-ligand interaction profiler to DNA and RNA. Nucleic Acids Res..

[34] Massey AJ (2018). A high content, high throughput cellular thermal stability assay for measuring drug-target engagement in living cells. PLoS One..

[35] Martinez Molina D, Jafari R, Ignatushchenko M, Seki T, Larsson EA, Dan C, Sreekumar L, Cao Y, Nordlund P (2013). Monitoring drug target engagement in cells and tissues using the cellular thermal shift assay. Science..

[36] Cimmperman P, Baranauskiene L, Jachimoviciūte S, Jachno J, Torresan J, Michailoviene V, Matuliene J, Sereikaite J, Bumelis V, Matulis D (2008). A quantitative model of thermal stabilization and destabilization of proteins by ligands. Biophys J..

[37] Leung SWS, Shi Y (2022). The glycolytic process in endothelial cells and its implications. Acta Pharmacol Sin..

[38] Li J, Li S, Guo J, Li Q, Long J, Ma C, Ding Y, Yan C, Li L, Wu Z, Zhu H, Li KK, Wen L, Zhang Q, Xue Q, Zhao C, Liu N, Ivanov I, Luo M, Xi R, Long H, Wang PG, Chen Y (2018). Natural product Micheliolide (MCL) irreversibly activates pyruvate kinase M2 and suppresses leukemia. J Med Chem..

[39] Pancholi V (2001). Multifunctional alpha-enolase: its role in diseases. Cell Mol Life Sci..

[40] Dalby A, Dauter Z, Littlechild JA (1999). Crystal structure of human muscle aldolase complexed with fructose 1,6-bisphosphate: mechanistic implications. Protein Sci..

[41] Vakifahmetoglu-Norberg H, Ouchida AT, Norberg E (2017). The role of mitochondria in metabolism and cell death. Biochem Biophys Res Commun..

[42] Subramaniam SR, Chesselet MF (2013). Mitochondrial dysfunction and oxidative stress in Parkinson's disease. Prog Neurobiol..

[43] Bell SM, Burgess T, Lee J, Blackburn DJ, Allen SP, Mortiboys H. Peripheral glycolysis in neurodegenerative diseases. Int J Mol Sci. 2020;21(23) 10.3390/ijms21238924 PMC7727792 33255513

[44] Mergenthaler P, Lindauer U, Dienel GA, Meisel A (2013). Sugar for the brain: the role of glucose in physiological and pathological brain function. Trends Neurosci..

[45] Zhang S, Lachance BB, Mattson MP, Jia X (2021). Glucose metabolic crosstalk and regulation in brain function and diseases. Prog Neurobiol..

[46] Tang BL (2020). Glucose, glycolysis, and neurodegenerative diseases. J Cell Physiol..

[47] Di Lazzaro G, Magrinelli F, Estevez-Fraga C, Valente EM, Pisani A, Bhatia KP (2021). X-linked parkinsonism: phenotypic and genetic heterogeneity. Mov Disord..

[48] Chen X, Zhao C, Li X, Wang T, Li Y, Cao C, Ding Y, Dong M, Finci L, Wang JH, Li X, Liu L (2015). Terazosin activates Pgk1 and Hsp90 to promote stress resistance. Nat Chem Biol..

[49] Cai R, Zhang Y, Simmering JE, Schultz JL, Li Y, Fernandez-Carasa I, Consiglio A, Raya A, Polgreen PM, Narayanan NS, Yuan Y, Chen Z, Su W, Han Y, Zhao C, Gao L, Ji X, Welsh MJ, Liu L (2019). Enhancing glycolysis attenuates Parkinson's disease progression in models and clinical databases. J Clin Invest..

[50] Wei Y, Lu M, Mei M, Wang H, Han Z, Chen M, Yao H, Song N, Ding X, Ding J, Xiao M, Hu G (2020). Pyridoxine induces glutathione synthesis via PKM2-mediated Nrf2 transactivation and confers neuroprotection. Nat Commun..

[51] Zhou Q, Tang M, He L, Chen S (2020). PKM2: a crucial neuroprotective target against oxidative stress. Acta Biochim Biophys Sin Shanghai..

[52] Jin J, Li GJ, Davis J, Zhu D, Wang Y, Pan C, Zhang J (2007). Identification of novel proteins associated with both alpha-synuclein and DJ-1. Mol Cell Proteomics..

[53] Poon HF, Frasier M, Shreve N, Calabrese V, Wolozin B, Butterfield DA (2005). Mitochondrial associated metabolic proteins are selectively oxidized in A30P alpha-synuclein transgenic mice--a model of familial Parkinson's disease. Neurobiol Dis..

[54] Dukes AA, Van Laar VS, Cascio M, Hastings TG (2008). Changes in endoplasmic reticulum stress proteins and aldolase a in cells exposed to dopamine. J Neurochem..

[55] Haddad F, Sawalha M, Khawaja Y, Najjar A, Karaman R. Dopamine and levodopa prodrugs for the treatment of Parkinson's disease. Molecules. 2017;23(1) 10.3390/molecules23010040 PMC5943940 29295587

[56] Masato A, Bubacco L, Greggio E (2021). Too much for your own good: excessive dopamine damages neurons and contributes to Parkinson's disease: an editorial highlight for "enhanced tyrosine hydroxylase activity induces oxidative stress, causes accumulation of autotoxic catecholamine metabolites, and augments amphetamine effects in vivo". J Neurochem..

