# Supplementary material for: Dopamine modification of glycolytic enzymes impairs glycolysis: possible implications for Parkinson’s disease
Source: Cell Commun Signal. 2024 Jan 29;22:75. doi: 10.1186/s12964-024-01478-0 (PMC10823740; doi:10.1186/s12964-024-01478-0)
Supplement: Supplementary file 1 — Additional file 1: Fig. S1. 1H NMR and 13C NMR of dopamine probe (DA-P). Fig. S2. CESTA and immunofluorescence to verify DA binding to PKM2 and aldolase A. A&B CETSA to verify DA binding to PKM2 or aldolase A. Cell lysates incubated with 100 µM DA for 2 h were subjected to CESTA-western blot analysis. C Representative fluorescence images of intracellular PKM2 in MN9D cells following labeling with 100 µM DA-P for 3 h. Error bars, mean ± s.d. Scale bar, 10 µm. *, p < 0.05. Fig. S3. DA suppresses the enzymatic activity of neuron α-enolase. A The immuno-blots showing the level of endogenous aldolase A after incubation with indicated concentrations of DA for 24 h. B Measurement of the catalytic activity of α-enolase extracted from cultured striatal neurons with the treatment of indicated concentrations of DA. C Measurement of the catalytic activity of α-enolase extracted from mouse midbrain tissue with the treatment of indicated concentrations of DA. Fig. S4. DA could bind to K230 residue of aldolase A, and Cys49 and Cys424 residues of PKM2. A-C In-gel fluorescence visualization of recombinant human aldolase A, α-enolase and PKM2 labeled by IAA-yne in the presence or absence of the competitors DA or IAA. D MS/MS spectrum of human aldolase A treated with 100 µM DA for 60 min. E A binding model of DA with human aldolase A predicted by molecular docking. F&G MS/MS spectrum of human PKM2 treated with 100 µM DA for 60 min. H A binding model of DA with human PKM2 predicted by molecular docking. Fig. S5. Metabolic dysfunction caused by DA via inducing mitochondrial damage. A-C Measurement of indicators of OCR rate in MN9D cells under different concentrations of DA (0, 100, 400 and 800 µM) treatment for 12 h. D Heatmap showing top up and down metabolites after 400 µM DA incubation for 12 h. E KEGG pathway enrichment analysis of DA-targeted metabolites. Top 25 pathways are shown. All error bars represent mean ± s.d. n.s. not significant; *, p < 0.05; **, p < 0.01; ***, p < 0.001; [file 12964_2024_1478_MOESM1_ESM.docx]

**
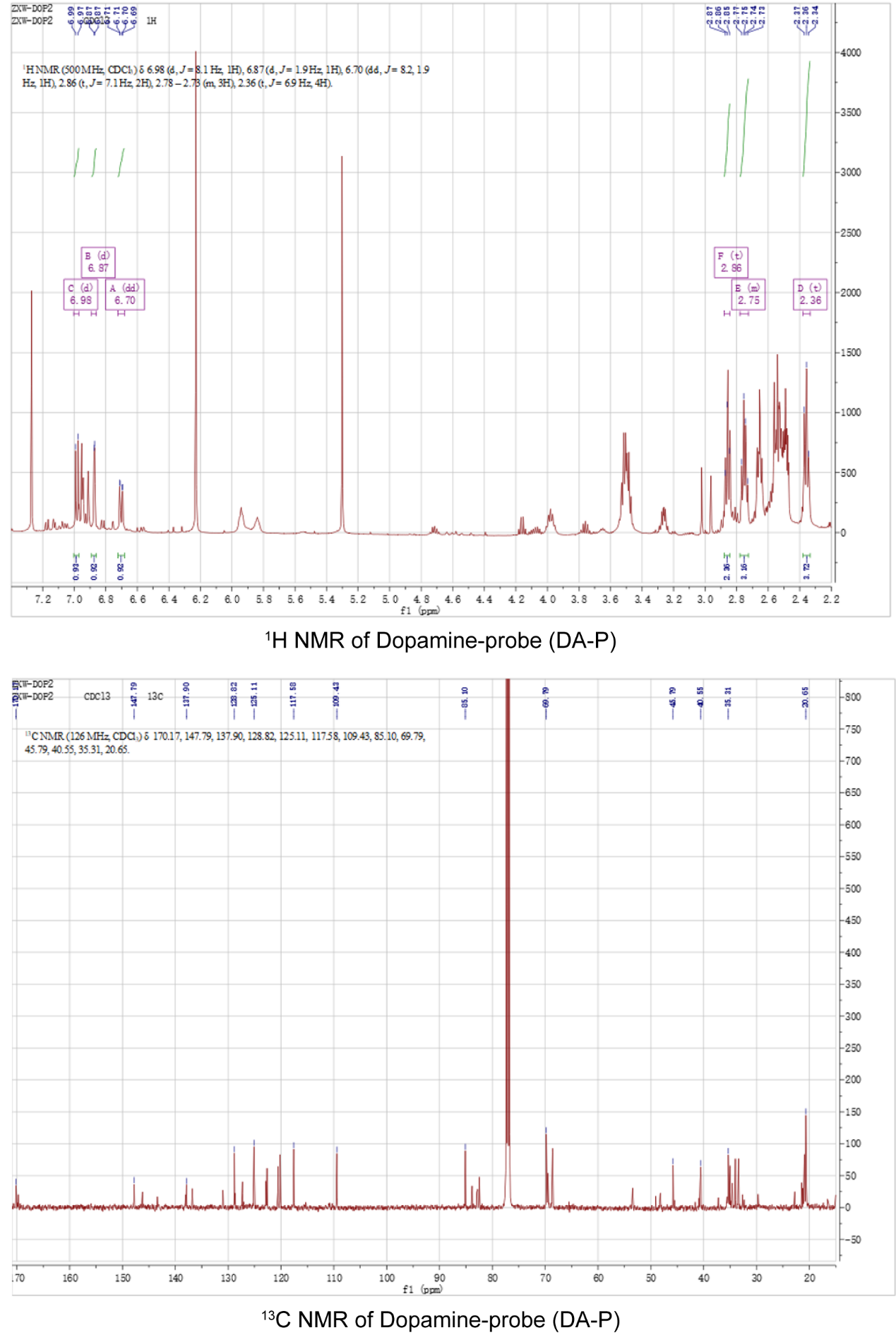
 Fig. S1.** ^1^H NMR and ^13^C NMR of dopamine probe (DA-P).

**
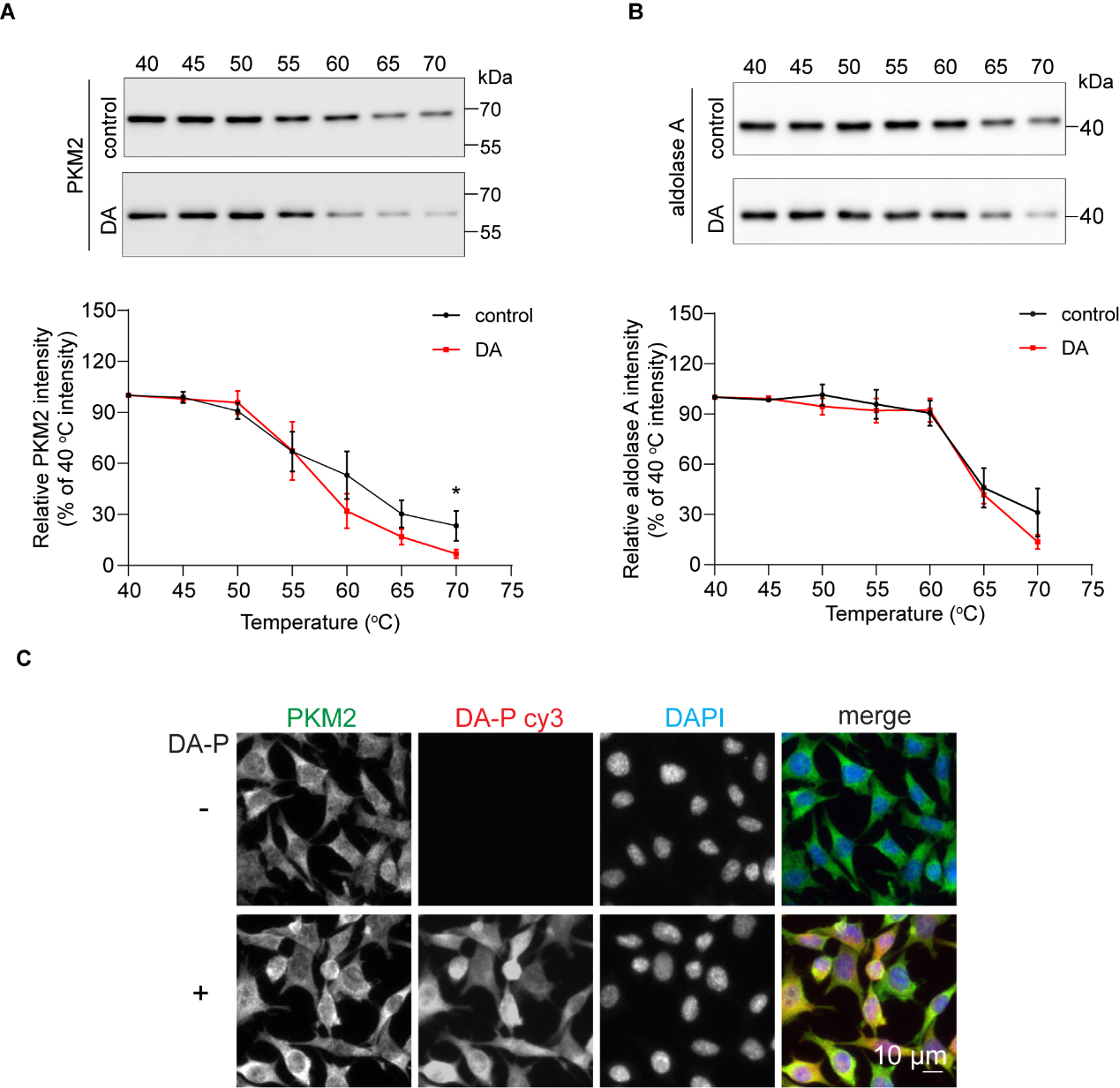
**

**Fig. S2.** CESTA and immunofluorescence to verify DA binding to PKM2 and aldolase A. **A&B** CETSA to verify DA binding to PKM2 or aldolase A. Cell lysates incubated with 100 µM DA for 2 h were subjected to CESTA-western blot analysis. **C** Representative fluorescence images of intracellular PKM2 in MN9D cells following labeling with 100 µM DA-P for 3 h. Error bars, mean ± s.d. Scale bar, 10 µm. *, *p* < 0.05.


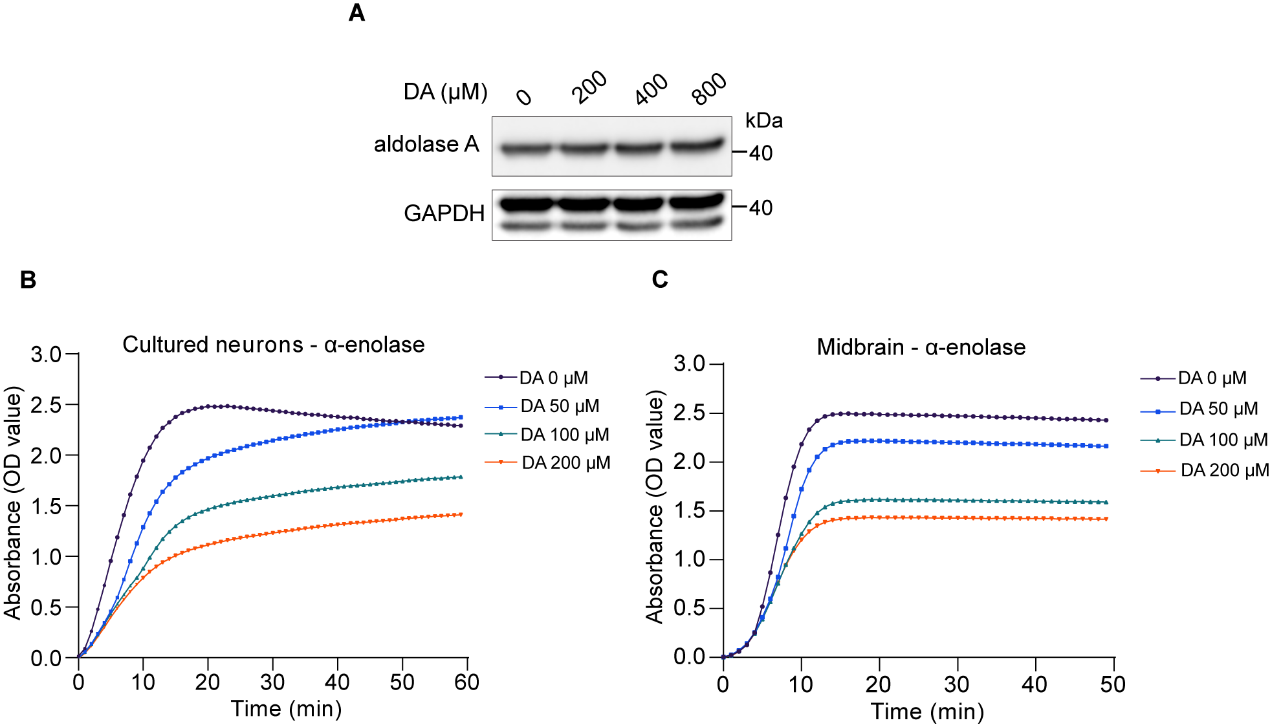


**Fig. S3.** DA suppresses the enzymatic activity of neuron α-enolase. **A** The immuno-blots showing the level of endogenous aldolase A after incubation with indicated concentrations of DA for 24 h. **B** Measurement of the catalytic activity of α-enolase extracted from cultured striatal neurons with the treatment of indicated concentrations of DA. **C** Measurement of the catalytic activity of α-enolase extracted from mouse midbrain tissue with the treatment of indicated concentrations of DA.

**
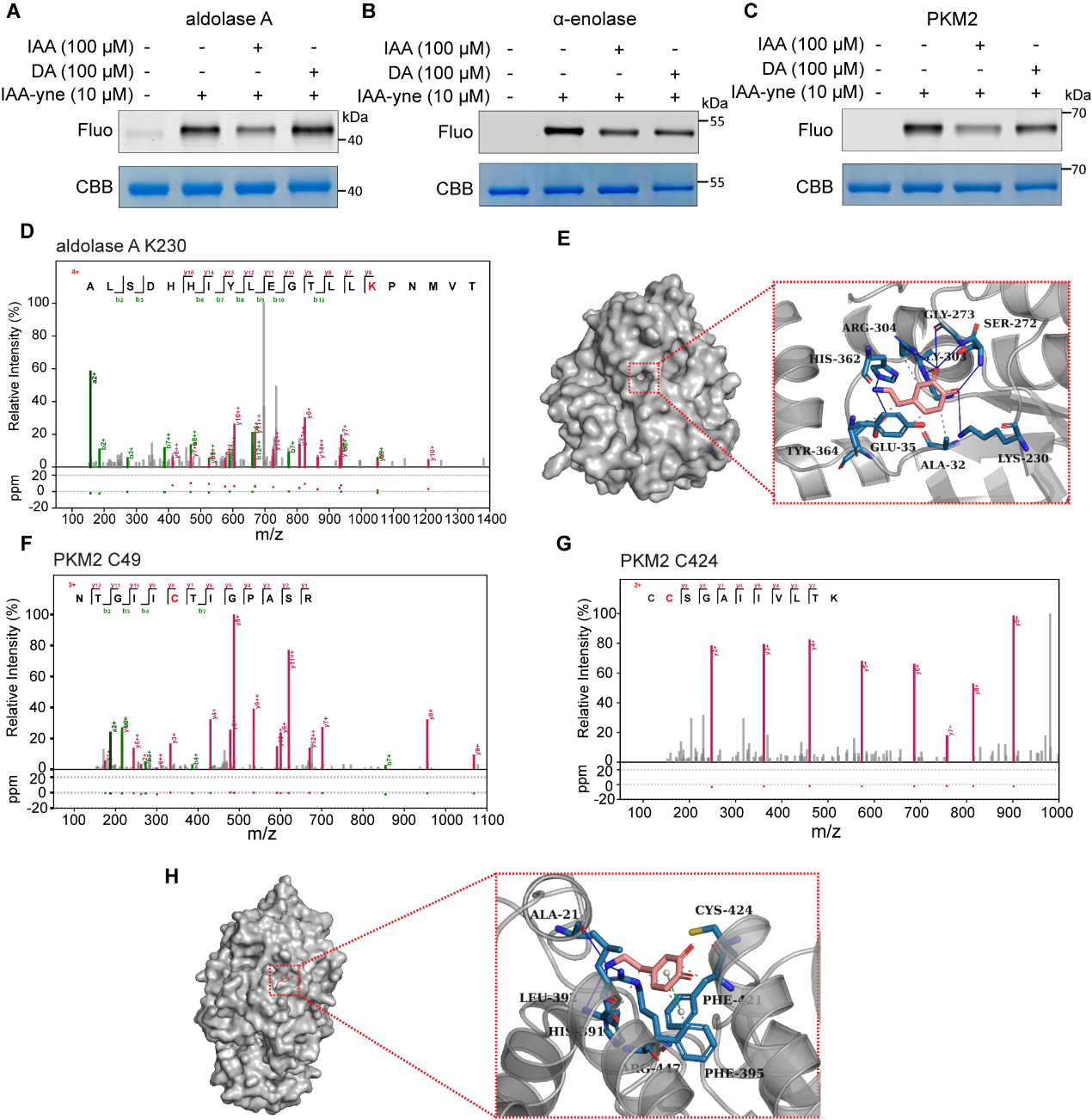
**

**Fig. S4.** DA could bind to K230 residue of aldolase A, and Cys49 and Cys424 residues of PKM2. **A-C** In-gel fluorescence visualization of recombinant human aldolase A, α-enolase and PKM2 labeled by IAA-yne in the presence or absence of the competitors DA or IAA. **D** MS/MS spectrum of human aldolase A treated with 100 µM DA for 60 min. **E** A binding model of DA with human aldolase A predicted by molecular docking. **F&G** MS/MS spectrum of human PKM2 treated with 100 µM DA for 60 min. **H** A binding model of DA with human PKM2 predicted by molecular docking.

**
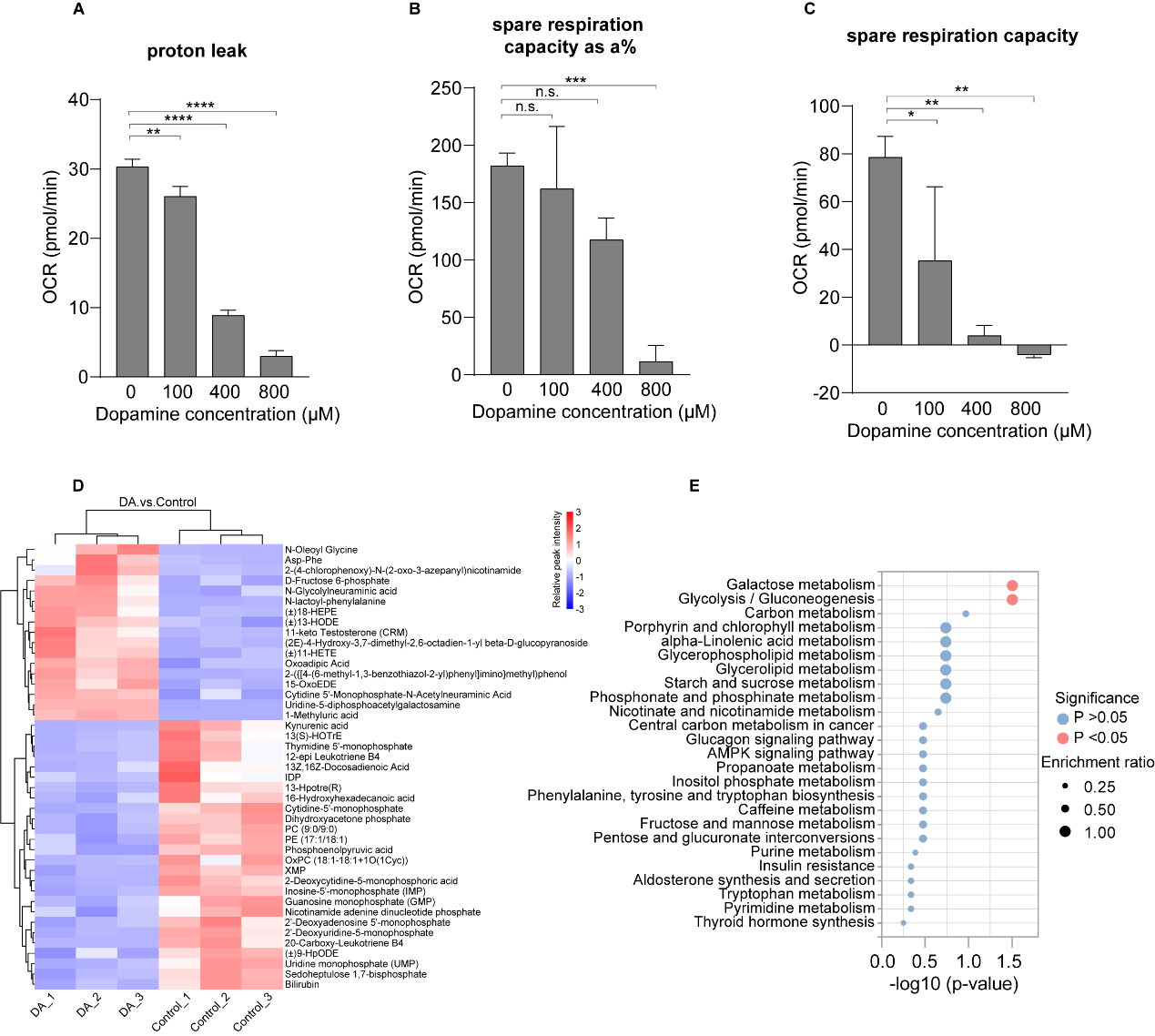
Fig. S5.** Metabolic dysfunction caused by DA via inducing mitochondrial damage. **A-C** Measurement of indicators of OCR rate in MN9D cells under different concentrations of DA (0, 100, 400 and 800 µM) treatment for 12 h. **D** Heatmap showing top up and down metabolites after 400 µM DA incubation for 12 h. **F** KEGG pathway enrichment analysis of DA-targeted metabolites. Top 25 pathways are shown. All error bars represent mean ± s.d. n.s. not significant; *, p < 0.05; **, p < 0.01; ***, p < 0.001; ****, p < 0.0001.
